# Effectiveness and Safety of Direct Oral Anticoagulants Versus Warfarin in Patients with Atrial Fibrillation and Cancer: A Target Trial Emulation from SEER-Medicare Database

**DOI:** 10.1007/s10557-024-07589-7

**Published:** 2024-06-07

**Authors:** Bang Truong, Lori Hornsby, Brent Fox, Chiahung Chou, Jingyi Zheng, Jingjing Qian

**Affiliations:** 1 Department of Health Outcomes Research and Policy, Auburn University Harrison College of Pharmacy, 4306d Walker Building, Auburn, AL USA; 2 Department of Pharmacy Practice, Auburn University Harrison College of Pharmacy, Auburn, AL USA; 3 Department of Mathematics and Statistics, Auburn University College of Sciences and Mathematics, Auburn, AL USA

**Keywords:** DOACs, Warfarin, AFib, Cancer, Stroke, Bleeding

## Abstract

**Background:**

Direct oral anticoagulants (DOACs) are preferred over warfarin in patients with atrial fibrillation (AFib). However, their safety and effectiveness in patients with AFib and cancer are inconclusive.

**Methods:**

We conducted a retrospective cohort study by emulating a target trial. Patients with a record of cancer (breast, prostate, or lung), newly diagnosed with AFib initiated DOACs or warfarin within 3 months after AFib diagnosis from the 2012–2019 Surveillance, Epidemiology, and End Results (SEER)-Medicare database were included. We compared the risk of ischemic stroke, major bleeding, and secondary outcomes (venous thromboembolism, intracranial bleeding, gastrointestinal bleeding, and non-critical site bleeding) between patients who initiated DOACs and warfarin. Inverse probability treatment weights and inverse probability censoring weights were used to adjust imbalanced patient and disease characteristics and loss to follow-up between the two groups. Weighted pooled logistic regression were used to estimate treatment effect with hazard ratios (HRs) with 95% confidence interval (95% CIs).

**Results:**

The incidence rates of stroke and major bleeding between DOAC and warfarin initiators were 9.97 vs. 9.91 and 7.74 vs. 9.24 cases per 1000 person-years, respectively. In adjusted intention-to-treat analysis, patients initiated DOACs had no statistically significant difference in risk of ischemic stroke (HR = 0.87, 95% CI 0.52–1.44) and major bleeding (HR = 1.14, 95% CI 0.77–1.68) compared to those initiated warfarin. In adjusted per-protocol analysis, there was no statistical difference in risk of ischemic stroke (HR = 1.81, 95% CI 0.75–4.36) and lower risk for major bleeding, but the 95% CI was wide (HR = 0.35, 95% CI 0.12–0.99) among DOAC initiators compared to warfarin initiators. The benefits in secondary outcomes were in favor of DOACs. The findings remained consistent across subgroups and sensitivity analyses.

**Conclusion:**

DOACs are safe and effective alternatives to warfarin in the management of patients with AFib and cancer.

**Supplementary Information:**

The online version contains supplementary material available at 10.1007/s10557-024-07589-7.

## Introduction

Atrial fibrillation (AFib) is the most common type of cardiac arrhythmia [1]. In the United States (US), AFib affects 2.7–6.1 million Americans and is associated with more than 454,000 hospitalizations and 158,000 deaths each year [2–4].

Oral anticoagulants (OACs) including warfarin and direct oral anticoagulants (DOACs) are approved for the management of AFib patients [5]. In the general AFib population, DOACs are associated with lower risk of ischemic stroke and bleeding compared with warfarin [6]. However, in the context of malignancy, AFib is associated with higher burden of mortality and mortality since both cancer and AFib are independent risk factors of ischemic stroke and venous thromboembolism (VTE) [7, 8]. Specifically, compared to patients with AFib, those with concomitant AFib and cancer have a fourfold to sevenfold higher risk of VTE and a twofold higher risk of bleeding [7, 8]. However, the effectiveness and safety profiles of DOACs and warfarin have not been well established among patients with AFib and cancer. Current guidelines from American Heart Association (AHA), European Society of Cardiology (ESC), American Society of Clinical Oncology (ASCO), and the International Society on Thrombosis and Haemostasis (ISTH) do not recommend any OAC over another [9–12]. As a result, less than half of AFib or atrial flutter patients with cancer initiated OACs [13–15].

Early randomized controlled trials (RCTs) comparing DOACs and warfarin in AFib patients excluded cancer patients due to potential drug-drug interactions with chemotherapy and low life expectancy (e.g., ENGAGE, ROCKET-AF, and ARISTOLE) [16–18]. A meta-analysis of these RCTs was conducted in subgroups of patients who developed cancer after randomization revealed that DOACs were associated with non-inferior rates of thromboembolic and bleeding events and reduced risk of VTE [19]. However, interpretation of the meta-analysis may be limited by heterogeneity in study design, cancer type, and treatment among included studies [19]. Recent observational studies comparing DOACs and warfarin among patients AFib and cancer have shown consistent findings compared with RCTs in favor of DOACs [20–28]. However, these observational studies were prone to limitations such as unmeasured confounding (cancer characteristics such as cancer stage, tumor grade, and cancer treatment). In addition, safety and effectiveness were rarely reported across cancer characteristics although they are important for tailoring the treatment selection in patients with cancer [20, 21, 26, 29].

In this study, we implemented a target trial framework to compare the effectiveness and safety profiles of DOACs and warfarin among newly diagnosed AFib patients with cancer using the Surveillance, Epidemiology, and End Results (SEER) registry linked to Medicare claims data to further capture cancer characteristics. The target trial emulation framework articulates the causal questions similar to an RCT protocol and explicitly emulating the components of that protocol using the observational data, which improves the quality of observational studies [30, 31].

## Materials and Methods

### Study Design and Data Source

We followed the Strengthening The Reporting of Observational studies in Epidemiology (STROBE) to report the components of our study [32]. We conducted a retrospective, population-based cohort study on the target trial framework [33, 34] using SEER registry linked to Medicare database from 2011 to 2019. SEER covers 48% of US population, collects, and releases data on cancer patients such as demographics, cancer characteristics, of treatment, and follow-up [35]. The linkage to Medicare data adds to SEER health care services utilization of beneficiaries [36]. The protocol for a target trial and emulation procedure is described in Table 1. The study design and timeline are illustrated in Figure S1.

**Table 1:** Protocol for a target trial and emulation procedure using the SEER-Medicare data base to estimate the risks of stroke and bleeding between warfarin users and DOAC users

Protocol component	Hypothetical target trial	Emulation in SEER-Medicare data
Eligibility criteria	▪ Patients aged ≥ 66, newly diagnosed with non-valvular AFib within 12 months before enrollment and history or active breast, lung, prostate cancer between January 1, 2012, and December 31, 2019 ▪ Beneficiaries continuously enrolled in Medicare part A, B, D, and without Medicare Advantage for 12 months before the diagnosis ▪ Baseline CHA_2_DS_2_-VASc score ≥ 2 ▪ No history of OAC use ▪ No history of mitral valve disease, heart valve repair or replacement, deep vein thrombosis, pulmonary embolism, or joint replacement ▪ Without any diagnosis of stroke within the previous 14 days ▪ Without any conditions associated with an increased risk of bleeding, including major surgery within the previous month, history of intracranial, intraocular, spinal, retroperitoneal or atraumatic intra‐articular bleeding, and gastrointestinal hemorrhage within the last 30 days ▪ Without renal impairment stage 5 or end-stage renal diseases within the last 12 months	Same as target trial
Treatment strategies	Eligible individuals are randomly assigned to warfarin or DOACs within a grace period of 3 months	We assume that study participants are randomly assigned conditioning on baseline covariates
Follow-up	The follow-up of target trials starts at the earliest time when treatment could be initiated, that is when the patients actually started their treatment within a grace period. End of follow-up is the occurrence of a specific study outcome, the end of administrative censoring (12 months after baseline), death, loss to follow-up, or December 31, 2019, whichever came first	Same as target trial
Outcomes	Primary outcomes: ischemic stroke, major bleeding Secondary outcomes: VTE, intracranial bleeding, gastrointestinal bleeding, and non-critical site bleeding	Same as target trial
Causal contrast	Intention-to-treat effect, per-protocol effect	Observational analog of intention-to-treat and per-protocol effect

*AFib* atrial fibrillation; *OAC* oral anticoagulant; *SEER* The Surveillance, Epidemiology, and End Results; *DOAC* direct oral anticoagulant; *VTE* venous thromboembolism; *CHA*_*2*_*DS*_*2*_*-VASc A* composite score for risk of stroke

### Study Sample and Eligibility Criteria

#### Study Sample

The study sample included individuals aged ≥ 66 with new onset non-valvular atrial fibrillation (NVAF) between January 1, 2012, and December 31, 2019, defined as any International Classification of Disease-9th Revision-Clinical Modification (ICD-9-CM) codes 427.31 or 427.32 or any International Classification of Disease-10th Revision-Clinical Modification (ICD-10-CM) codes I48.xx in any position on one Medicare inpatient claim or on two outpatient claims at least 7 days but < 1 year apart (Table S1**, **Supplemental material**s**) [37]. We retained new NVAF patients with any record of breast (ICD-O-3 codes C50.0-C50.9), lung (ICD-O-3 codes C34.0, C34.1, C34.2, C34.3, C34.8, C34.9, C33.9), and prostate cancer (ICD-O-3 codes C61.9) — the most commonly concomitant cancer types in AFib [21, 38, 39] in SEER files at any time before the initial AFib diagnosis. We required patients to continuously enroll in Medicare parts A, B, D, and without Medicare Advantage or Health Maintenance Organization (HMO) for 12 months before AFib diagnosis. We furthered restrict the study sample to those with a moderate to high risk of stroke, defined as CHA_2_DS_2_-VASc score ≥ 2 [40, 41].

#### Exclusion Criteria

We adapted exclusion criteria based on RE-LY and ROCKET-AF trials [40, 41]. In brief, we removed participants with indications for warfarin or DOACs other than NVAF or absolute contra-indications of warfarin or DOACs such as (1) any OAC use during the 12 months baseline period; (2) presence of mitral valve disease, heart valve repair or replacement, deep vein thrombosis, pulmonary embolism, or joint replacement during the 12 months baseline period; (3) any stroke within 14 days before first NVAF diagnosis; (4) major surgery (i.e., hip fracture, cardiac surgery), intracranial, intraocular, spinal, retroperitoneal or atraumatic intra‐articular bleeding, and gastrointestinal hemorrhage within 30 days before AFib diagnosis; (5) renal impairment stage 5 or end-stage renal diseases, during the 12 months baseline period. Individuals with any outcome of interest occurred before OAC initiation were also excluded from the analysis. All ICD codes to identify these conditions can be found in Table S1,Supplemental material.

### Treatment Strategies and Assignments

In the hypothetical target trial, eligible individuals were randomly assigned to either (1) warfarin or (2) DOACs and continued the assigned treatment during follow-up. In the emulation of target trial, we assumed randomization was attained given baseline covariates [42]. OAC prescriptions (including warfarin and dabigatran, apixaban, rivaroxaban, edoxaban) were identified from Medicare Part D Prescription Drug Event (PDE) files using NDC, regardless of OAC dosage [43]. We used a grace period of 3 months to determine the treatment group of eligible individuals since a 3-month grace period has been specified in previous RCTs and the risk of stroke in general AFib patients is highest in the first 3 months after new AFib diagnosis [44, 45]. OAC discontinuation was defined as a gap in OAC prescription for ≥ 30 days from the last day of days’ supply in PDE file.

### Follow-up

The follow-up started when the patients received the treatment within a grace period and ended at the occurrence of a specific study outcome, administrative censoring (12 months after baseline), death (all-cause deaths from SEER and Medicare files via the variables of “Date of Death Flag”), loss to follow-up (the earliest of 30 days after the end of continuous Medicare part A, B, or D enrollment or enrollment in an HMO), or December 31, 2019, whichever came first. For *per-protocol* analyses, follow-up also ends when the observed treatment deviated from initial treatment. Specifically, eligible individuals who discontinued the assigned treatment or switched their assigned treatment during follow-up (i.e. switching from warfarin to DOACs or vice versa) were censored.

### Outcomes

The primary effectiveness and safety outcomes were ischemic stroke and major bleeding, respectively. We defined major bleeding based on the bleeding site, according to the algorithm by previously developed algorithms (i.e., intraarticular, intracranial, intramuscular, intraocular, intraspinal, pericardial, and retroperitoneal), identified by ICD-9-CM and ICD-10-CM codes in the primary diagnosis from Medicare medical claims files [21, 46, 47]. Secondary outcomes such as VTE, intracranial bleeding, gastrointestinal (GI) bleeding, and other non-critical site bleeding were defined by ICD-9-CM and ICD-10-CM codes from Medicare inpatient claims using validated algorithms [46–48].

### Covariates

We carefully selected covariates based on published RCTs and observational studies [21, 38, 40]. Baseline covariates were extracted within 12-month period prior to first AFib diagnosis, including *demographics* (index age, sex, race/ethnicity, calendar year, geographical region, urbanicity), *socioeconomic factors* (household median income, percentage of household below poverty level, education level, Medicaid eligibility), *risk score* (CHA_2_DS_2_-VASc, HAS-BLED, and Comorbidity Scores SEER-Medicare version 2021 (NCI) [49]), *individual comorbidities* (asthma/chronic obstructive pulmonary disease, hematological disorders, dementia, depression, thrombocytopenia, acute kidney disease (AKD), peptic ulcer disease (PUD)), *cancer characteristics* (time from cancer diagnosis to the onset of AFib, cancer stage, tumor grade, cancer type, active cancer status [21, 38]), *cancer treatment* (radiation, and cancer-directed surgery, and potentially interacting antineoplastic agents), and *medication history* (angiotensin-converting enzyme inhibitors (ACEIs)/angiotensin II receptor blockers (ARBs), calcium channel blockers (CCB), beta blockers, antiarrhythmic medications, diuretics, statin, pump proton inhibitors, and serotonin reuptake inhibitors). Socioeconomic factors such as household income and education level are available at the aggregate area level. If patients had more than one type of cancer, we retained the most recent cancer diagnosis. Cancer treatment was obtained from diagnosis codes or procedures codes within 30 days before NVAF diagnosis. Other cancer characteristics such as number of regional nodes examined, tumor size, TNM classification, and other cancer-type specific characteristics such as hormone receptor status (HR), and human epidermal growth factor receptor 2 (HER2) for breast cancer or histologic type for lung cancer were used for descriptive purpose but not adjusted in the analysis due to high proportion of missing values [50].

For *per-protocol* analysis, the following time-varying covariates were extracted at a monthly basis after OAC initiations, including CHA_2_DS_2_-VASc score, HAS-BLED score, thrombocytopenia, AKD, radiation, cancer-directed surgery, and use of interacting treatment. All diagnosis codes and procedure codes for covariate ascertainment are described in Table S1, Supplementary materials. We used multiple imputation algorithm (fully conditional specification with logistic regression for categorical variables and predictive mean matching for continuous variables) to impute missing values [51].

### Causal Contrast

We computed the observational analog of both *intention-to-treat* (ITT) and *per-protocol* (PP) effects. ITT effect refers to effect of being assigned to warfarin or DOACs, regardless of whether individuals adhere to initial strategies during follow-up [52, 53]. For PP effect, those who were assigned to warfarin group were censored if they discontinued warfarin or switched from warfarin to DOACs and vice versa.

### Statistical Analysis

Descriptive statistics with mean and standard deviation (SD) for continuous variables, frequency count and percentage for categorical variables were used to describe the study sample. In the main analysis, we obtained the ITT effect (the effect of initiating DOACs compared with warfarin) and PP effect (the effect of sustaining DOACs compared with warfarin). First, we adjusted for potential confounders between treatment groups at each month using weighting approach. The total weights were a product of stabilized inverse probability treatment weights (IPTWs) and stabilized inverse probability censoring weights (IPCWs) due to loss to follow-up. Next, we fitted a weighted pooled logistic regression estimated by generalized estimating equations (GEEs) with robust variance estimators, adjusted for baseline and time-varying confounders. Incidence rate (case per person-year), absolute risk difference (RD, 95% CIs), and summary hazard ratios with 95% confidence intervals (HR, 95% CIs), and weighted survival curves were obtained. All weights were truncated at 99th percentile. The technical details of the adjustment are described in Technical Appendix. Statistical analysis was conducted using SAS (version 9.4, SAS Institute Inc., Cary, NC, USA).

### Subgroup Analyses and Sensitivity Analyses

Treatment effects were estimated under the following subgroups: cancer type (breast, lung, prostate), cancer status at baseline (active, history), cancer stage (local, regional, and distant), and tumor grade (I, II, and III). A series of sensitivity analyses were conducted to confirm the robustness of main findings. First, we extended a grace period to 6 months from AFib diagnosis to warfarin/DOAC initiation to reflect the uncertainty in clinical practice when patients are allowed time to complete clinical tests before treatment initiation or get access to initial treatment. Second, we included individuals with all levels of baseline CHA_2_DS_2_-VASc score because patients with cancer are at higher risk of stroke and may be eligible to initiate OACs. Third, we estimated the long-term treatment effects by extending follow-up time to 36 months. Fourth, we removed individuals with metastatic cancer at baseline, who may have a low life expectancy based on previous RCTs [40, 41]. Fifth, we excluded individuals with thrombocytopenia at baseline, since these patients are at elevated risk of bleeding [29, 54]. Six, we further truncated stabilized weights at 95th percentile to test the robustness of the treatment effects to the presence of extreme weights.

## Results

### Study Sample and Characteristics

**Fig. 1:**
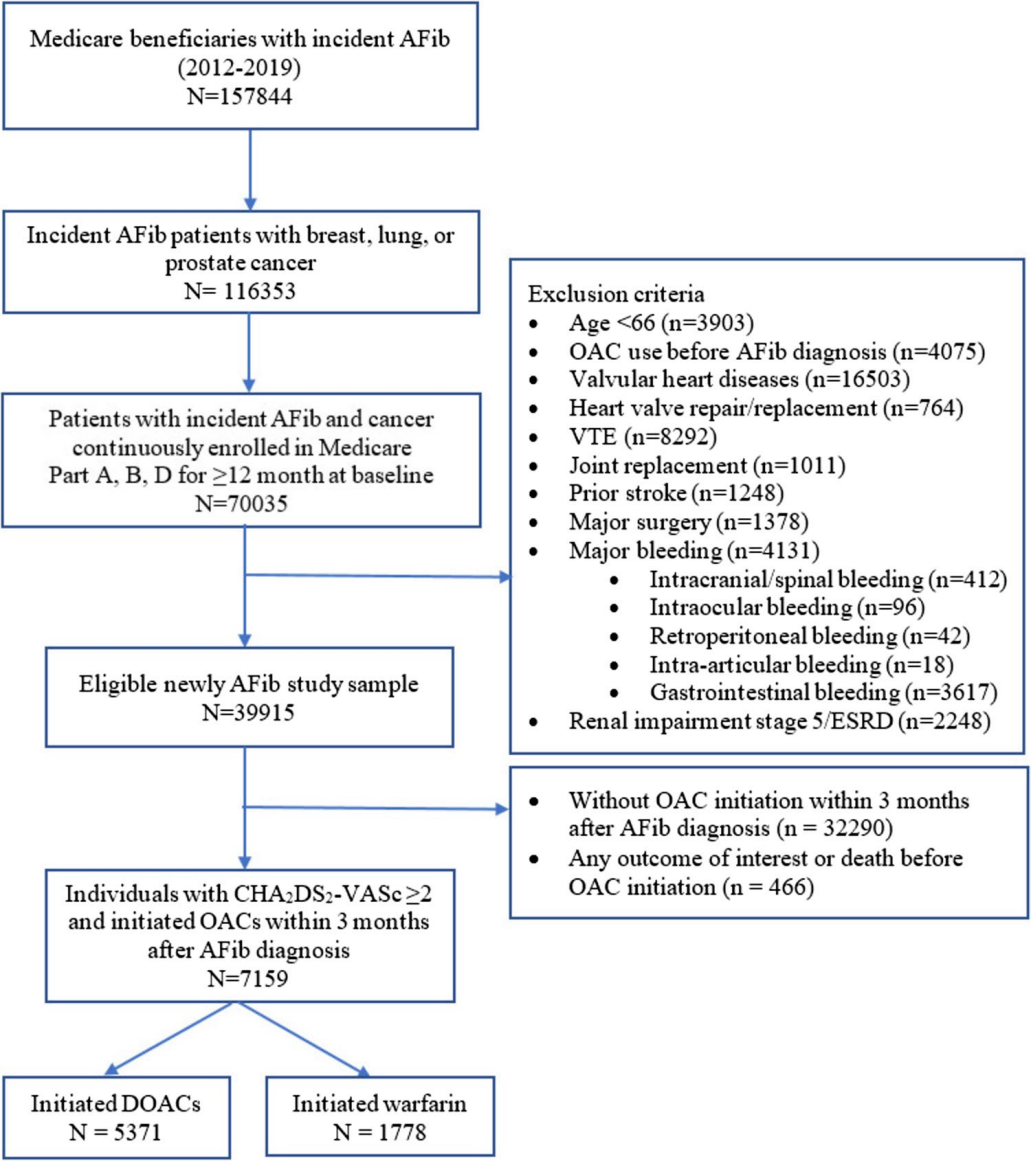
Flowchart diagram for study sample selection

Among 70,035 patients with newly diagnosis of AFib and concomitant cancer in SEER-Medicare 2012–2019, 5371 DOAC initiators (3264 apixaban, 314 dabigatran, 1786 rivaroxaban, seven edoxaban) and 1788 warfarin initiators were included in the final sample (Fig. 1).

Study sample characteristics are fully described in Table S2. Patients who initiated DOACs had a higher socio-economic status (higher household median income and education level) and lower comorbidity burden (CHA_2_DS_2_-VASc, HAS-BLED, and NCI scores) compared to those initiated warfarin. Regarding cancer characteristics, patients with breast or prostate cancer were more likely to receive DOACs while more patients with lung cancer were on warfarin. Warfarin was more commonly prescribed in patients with active cancer and patients in the advanced stage of cancer (regional and distant) (Table S2).

### Main Analysis

**Fig. 2:**
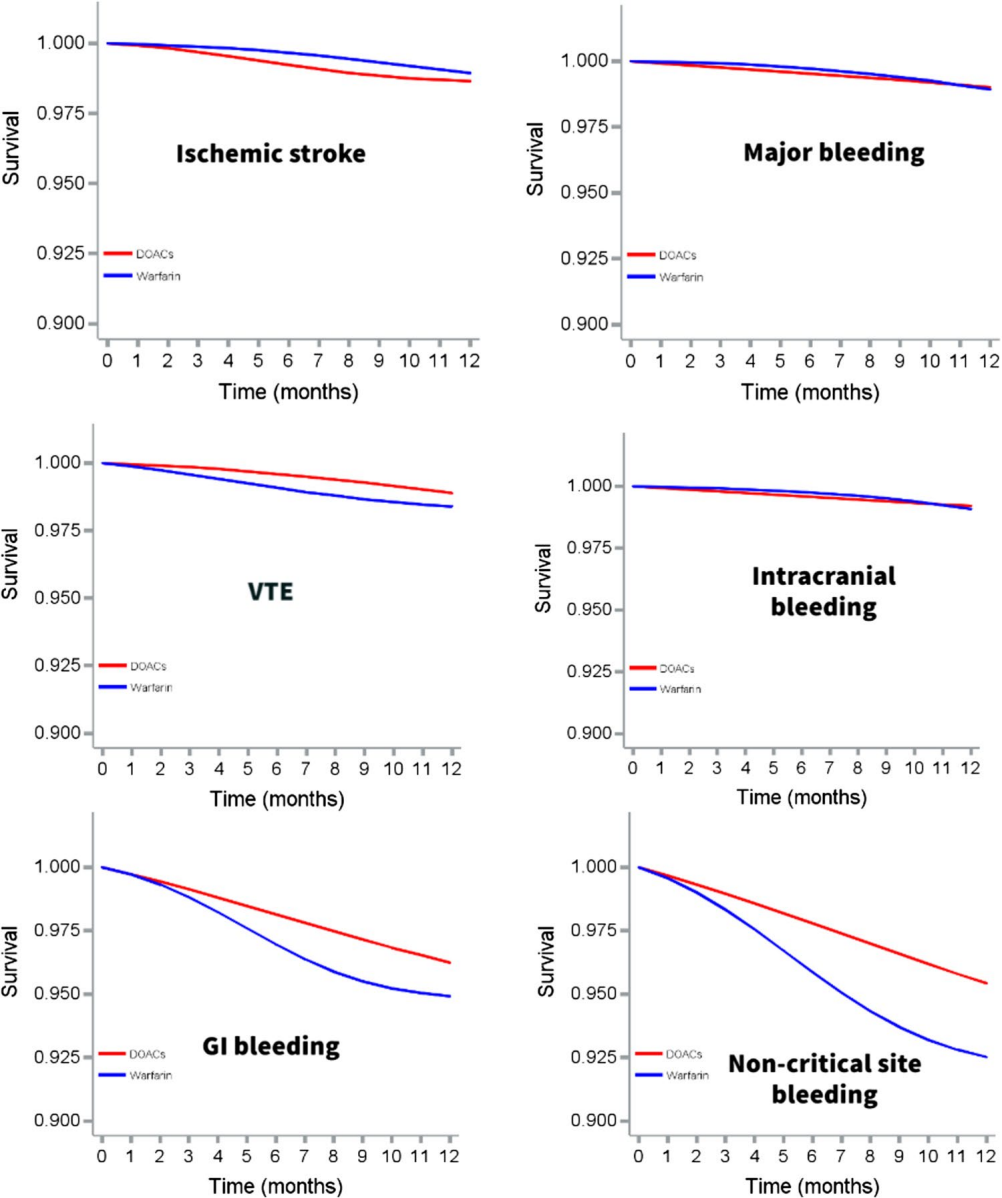
Weighted survival curves for intention-to-treat analysis comparing safety and effectiveness between direct oral anticoagulants and warfarin in patients with atrial fibrillation and cancer

**Fig. 3:**
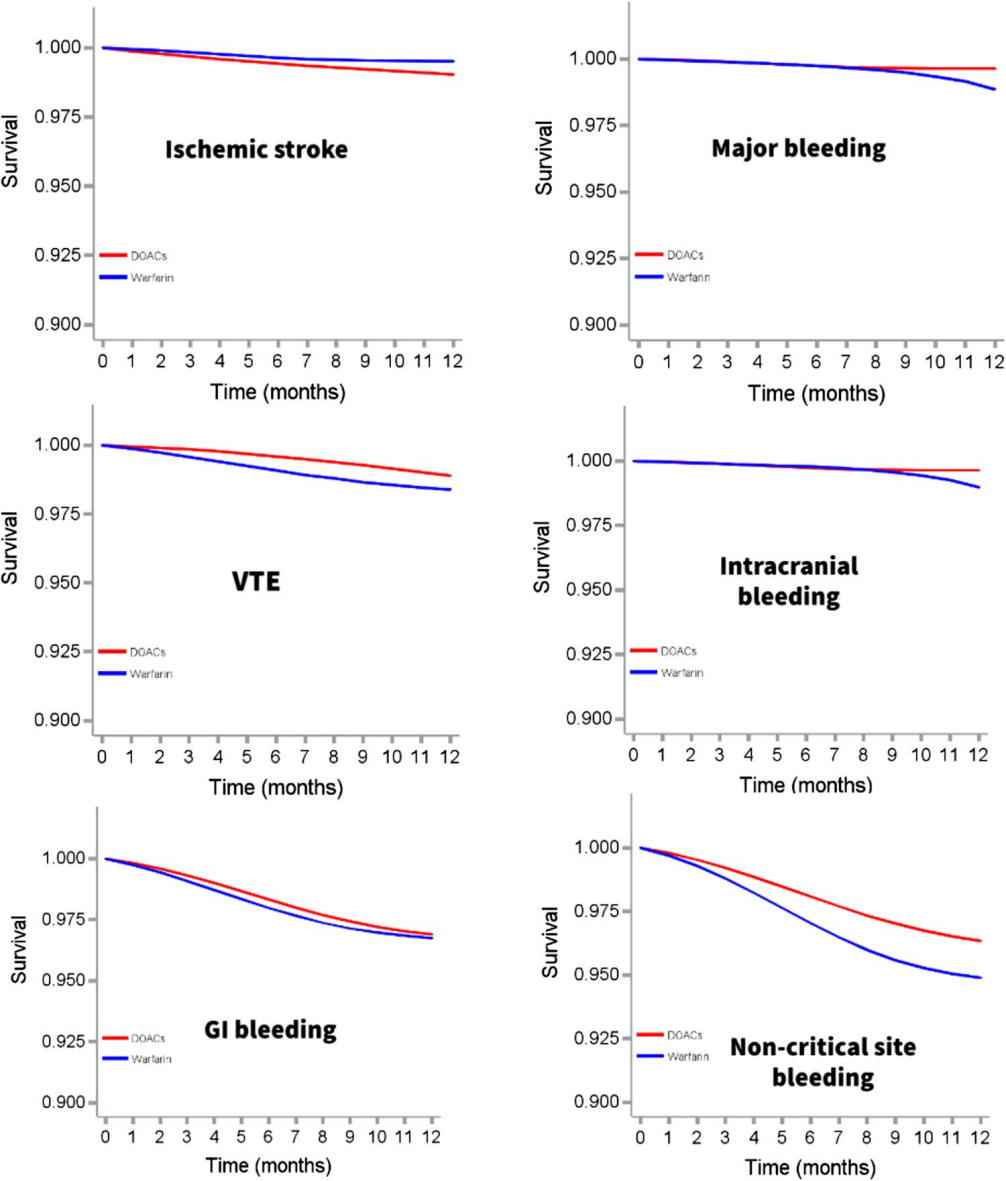
Weighted survival curves for per-protocol analysis comparing safety and effectiveness between direct oral anticoagulants and warfarin in patients with atrial fibrillation and cancer

The incidence rates of stroke and major bleeding between DOAC and warfarin initiators were 9.97 vs. 9.91 and 7.74 vs 9.24 cases per 1000 person-years, respectively. In adjusted *ITT analysis*, DOAC initiators had no statistically significant difference in risk of ischemic stroke (HR = 1.14, 95% CI 0.77–1.68) and major bleeding (HR = 0.87, 95% CI 0.52–1.44) as warfarin initiators. DOAC initiators also had no statistically significant difference in risk of VTE (HR = 0.73, 95% CI 0.47–1.15), and intracranial bleeding (HR = 0.78, 95% CI 0.45–1.35), but lower risk of GI bleeding (HR = 0.77, 95% CI 0.59–0.99) and non-critical site bleeding (HR = 0.63, 95% CI 0.50–0.77) compared to warfarin initiators (Fig. 2; Table 2). The findings were mostly consistent in adjusted *PP analysis* (ischemic stroke (HR = 1.81, 95% CI 0.75–4.36), VTE (HR = 0.50, 95% CI 0.20–1.09), intracranial bleeding (HR = 0.38, 95% CI 0.13–1.12), GI bleeding (HR = 0.91, 95% CI 0.61–1.35), and non-critical site bleeding (HR = 0.69, 95% CI 0.48–0.98)) (Fig. 3; Table 3). However, DOAC initiators had lower risk of major bleeding (HR = 0.35, 95% CI 0.12–0.99) than warfarin initiators in adjusted *PP analysis*, although 95% CI was wide. The distributions of total weights and truncated weights are described in Table S2.

**Table 2:** Intention-to-treat analysis for comparative effectiveness and safety between DOACs and warfarin in patients with AFib and cancer

	DOACs (*N* = 5371)		Warfarin (*N* = 1788)	
	Event (%)	Incidence rate*	Event (%)	Incidence rate *
Primary outcomes				
Stroke	45 (0.84)	9.97	15 (0.84)	9.91
Unadjusted RD (95% CI)	0.00% (− 0.49%, 0.49%)		Reference	
Unadjusted HR (95% CI)	1.00 (0.56, 1.80)		Reference	
Adjusted RD (95% CI)	0.02% (− 0.05%, 0.09%)		Reference	
Adjusted HR (95% CI)	1.14 (0.77–1.68)		Reference	
Major bleeding	35 (0.65)	7.74	14 (0.78)	9.24
Unadjusted RD (95% CI)	− 0.13% (− 0.59%, 0.33%)		Reference	
Unadjusted HR (95% CI)	0.84 (0.45, 1.55)		Reference	
Adjusted RD (95% CI)	− 0.02% (− 0.08%, 0.07%)		Reference	
Adjusted HR (95% CI)	0.87 (0.52–1.44)		Reference	
Secondary outcomes				
Venous thromboembolism	32 (0.60)	7.07	20 (1.2)	13.25
Unadjusted RD (95% CI)	− 0.52% (− 1.05%, 0.01%)		Reference	
Unadjusted HR (95% CI)	**0.53 (0.31, 0.93)**		Reference	
Adjusted RD (95% CI)	− 0.04% (− 0.13%, 0.05%)		Reference	
Adjusted HR (95% CI)	0.73 (0.47, 1.15)		Reference	
Intracranial bleeding	28 (0.52)	6.19	12 (0.67)	7.92
Unadjusted RD (95% CI)	− 0.15% (− 0.57%, 0.27%)		Reference	
Unadjusted HR (95% CI)	0.78 (0.40, 1.53)		Reference	
Adjusted RD (95% CI)	− 0.01% (− 0.06%, 0.04%)		Reference	
Adjusted HR (95% CI)	0.78 (0.45, 1.35)		Reference	
Gastrointestinal bleeding	153 (2.85)	34.24	62 (3.47)	41.71
Unadjusted RD (95% CI)	− 0.62% (− 1.58%, 0.34%)		Reference	
Unadjusted HR (95% CI)	0.82 (0.61, 1.10)		Reference	
Adjusted RD (95% CI)	** − 1.03% (− 2.25%, − 0.05%)**		Reference	
Adjusted HR (95% CI)	**0.77 (0.59, 0.99)**		Reference	
Non-critical site bleeding	185 (3.44)	41.53	88 (4.92)	59.64
Unadjusted RD (95% CI)	** − 1.48% (− 2.59%, − 0.36%)**		Reference	
Unadjusted HR (95% CI)	**0.69 (0.54, 0.90)**		Reference	
Adjusted RD (95% CI)	** − 1.78% (− 2.45%, − 1.11%)**		Reference	
Adjusted HR (95% CI)	**0.63 (0.50, 0.77)**		Reference	

^*^Number of events per 1000 person-years

*DOACs* direct oral anticoagulants, *RD* risk difference, *HR* hazard ratio, *CI* confidence interval

**Table 3:** Per-protocol analysis for comparative effectiveness and safety between DOACs and warfarin in patients with AFib and cancer

	DOACs (*N* = 5317)		Warfarin (*N* = 1788)	
	Event (%)	Incidence rate*	Event (%)	Incidence rate*
Primary outcomes				
Stroke	35 (0.65)	11.1	–	6.13
Unadjusted RD (95% CI)	0.32% (− 0.03, 0.66)		Reference	
Unadjusted HR (95% CI)	1.69 (0.75, 4.03)		Reference	
Adjusted RD (95% CI)	0.04% (− 0.01%, 0.09%)		Reference	
Adjusted HR (95% CI)	1.81 (0.75–4.36)		Reference	
Major bleeding	23 (0.43)	7.29	–	9.18
Unadjusted RD (95% CI)	− 0.08% (− 0.05%, 0.30%)		Reference	
Unadjusted HR (95% CI)	0.48 (0.21, 1.12)		Reference	
Adjusted RD (95% CI)	− 0.06% (− 0.14%, 0.02%)		Reference	
Adjusted HR (95% CI)	**0.35 (0.12, 0.99)**		Reference	
Secondary outcomes				
Venous thromboembolism	18 (0.34)	5.70	15 (0.84)	15.34
Unadjusted RD (95% CI)	** − 0.50% (− 0.95%, − 0.05%)**		Reference	
Unadjusted HR (95% CI)	0.46 (0.22, 1.00)		Reference	
Adjusted RD (95% CI)	− 0.04% (− 0.10%, 0.01%)		Reference	
Adjusted HR (95% CI)	0.50 (0.20, 1.09)		Reference	
Intracranial bleeding	18 (0.34)	5.70	–	8.16
Unadjusted RD (95% CI)	− 0.11% (− 0.46%, 0.23%)		Reference	
Unadjusted HR (95% CI)	0.51 (0.21, 1.23)		Reference	
Adjusted RD (95% CI)	− 0.05% (− 0.13%, 0.03%)		Reference	
Adjusted HR (95% CI)	0.38 (0.13, 1.12)		Reference	
Gastrointestinal bleeding	120 (2.23)	38.20	44 (2.46)	45.29
Unadjusted RD (95% CI)	− 0.23% (− 1.05%, 0.59%)		Reference	
Unadjusted HR (95% CI)	0.83 (0.57, 1.21)		Reference	
Adjusted RD (95% CI)	− 0.03% (− 0.14%, 0.08%)		Reference	
Adjusted HR (95% CI)	0.91 (0.61–1.35)		Reference	
Non-critical site bleeding	144 (2.68)	45.87	65 (3.64)	67.10
Unadjusted RD (95% CI)	− 0.95% (− 1.92%, 0.01%)		Reference	
Unadjusted HR (95% CI)	**0.69 (0.50, 0.95)**		Reference	
Adjusted RD (95% CI)	** − 0.94% (− 1.26%, − 0.62%)**		Reference	
Adjusted HR (95% CI)	**0.69 (0.48, 0.98)**		Reference	

–: suppressed due to cell size < 11; *: number of events per 1000 person-years

*DOACs* direct oral anticoagulants, *RD* risk difference, *HR* hazard ratio, *CI* confidence interval

### Subgroup and Sensitivity Analyses

The main findings were consistent with minor heterogeneity across subgroups of cancer type, active/inactive cancer status, cancer stage, and tumor grade. Due to small sample size and few events, subgroup analyses were not conducted for some outcomes in prostate cancer, regional or metastatic cancer, or tumor grade I. Statistically significant benefits of DOACs over warfarin were detected for VTE in breast cancer; GI bleeding and non-critical site bleeding in prostate cancer and local cancer stage; VTE and non-critical site bleeding in inactive cancer and tumor grade II; major bleeding and intracranial bleeding in patients with regional cancer stage and tumor grade III (Table S3). Finding remained robust under different assumption of grace period, additional inclusion and inclusion criteria, extended follow-up, and extreme weights (Table S4).

## Discussion

By explicitly emulating the target trial comparing effectiveness and safety between DOACs and warfarin, we found that DOAC initiators had no significant difference in risks of ischemic stroke and major bleeding compared with warfarin. However, DOAC initiators had lower risk of major bleeding than warfarin initiators in adjusted PP analysis. In addition, DOACs initiators also had lower risk of secondary outcomes including GI bleeding and non-critical site bleeding than warfarin initiators. Findings remain consistent across subgroups and robust in sensitivity analyses.

The management of patients with AFib and cancer are more complicated than general AFib patients because the presence of cancer increases the risk of stroke and bleeding in these patients [7, 18]. Although clinical guidelines are inconclusive, DOACs were increasingly preferred for the management of AFib in the presence of cancer [21, 28, 39]. The growing body of evidence in the literature showed inconsistent findings in comparative effectiveness and safety between DOACs and warfarin in patients with AFib and cancer. In RCTs such as ENGAGE, ROCKET-AF, and ARISTOLE, patients with existing cancer were generally excluded due to their low life expectancy [16–18]. A meta-analysis of these RCTs found that DOACs were associated with non-inferior rates of thromboembolic and bleeding events compared with warfarin [19]. A potential limitation of the meta-analysis is clinical heterogeneity (i.e., patient characteristics, treatment, follow-up) and methodological heterogeneity (i.e., study design and analysis) among included studies [19]. Using administrative claims data, Shah (2018) and Deitelzweig (2021) found similar risk of stroke and bleeding between DOACs and warfarin, except for apixaban (lower risk of stroke and bleeding) [21, 38]. Shah also found a lower risk of stroke among DOAC user compared with warfarin. An important limitation of these studies is unmeasured confounding, such as cancer stage and tumor grade (not available in claims data), which may result in a differential risk of stroke and bleeding between DOACs and warfarin. In addition, although DOACs are associated with reduced risk of intracranial bleeding in non-cancer patients [55, 56], the benefit over warfarin was not found in our study. Indeed, both ITT and PP analyses showed a trend of reduced intracranial bleeding for DOACs initiators than warfarin initiators (HR = 0.78 (0.45, 1.35) and HR = 0.38 (0.13, 1.12)), but the treatment effects were not statistically different. This may be due to the small number of events in both groups and the small sample sizes of this study. In addition, pharmacokinetic and pharmacokinetics of OACs in cancer patients may not be stable as in non-cancer patients due to hemodynamic changes or drug-drug interactions with antineoplastic agents, which may prevent OACs from achieving their therapeutic effects [17, 18, 57–59]. Recently, Mehta (2022) used the SEER-Medicare data and conducted a new-user cohort study comparing risk of stroke and bleeding between DOACs and warfarin to further capture cancer characteristics from SEER registry [28]. The authors found an increased risk of stroke (HR = 1.41, 95% CI 0.92–2.14) but decreased risk of bleeding (HR = 0.90, 95% CI 0.70–1.17) among DOACs users compared to warfarin users. Since Mehta included patients with existing AFib and prior stroke or recent bleeding before OAC initiation, some patients may take DOACs or warfarin for secondary prevention of stroke. As a result, the observed incidence rates of ischemic stroke and bleeding are higher than our study because prior stroke or bleeding are strong risk factors for subsequent events [60–63]. In the current study, we emulated a target trial to explicitly answer a causal question: among patients with existing cancer who were newly diagnosed with AFib, what is the effect of initiating and/or sustaining DOACs compared with warfarin? We required patients to be outcome-free shortly before AFib diagnosis and during the grace period before they actually received OACs. This approach ascertains unbiased estimates for effectiveness and safety of OACs for primary prevention purposes and better interpretability in our study. Other strengths of our study included the adjustment for selection bias due to loss to follow-up [64], and adjustment for time-varying covariates [65], and estimand of interest specification (ITT or PP) [66]. Our findings contribute to the growing evidence and are expected to help clinicians optimize anticoagulation therapy in patients with AFib and cancer.

Our study may not perfectly emulate components of hypothetical target trials and referent RCTs such as inclusion/exclusion criteria, outcomes, and follow-up [40, 41]. For instance, RCTs excluded patients platelet count < 90,000/μL, systolic blood pressure ≥ 180 mmHg or diastolic blood pressure ≥ 100 mmHg, or creatinine clearance less than 30 mL/min at the baseline [40, 41]. However, we failed to obtain these lab values in SEER-Medicare. As a solution, we defined these conditions by the presence of thrombocytopenia or severe renal impairments or ESRD. In addition, several conditions were evaluated by clinicians’ assessment in RCTs rather than ICD codes in medical records, such as AFib definition by an abnormal electrocardiogram (ECG) [40, 41]. Also, we could not use international normalized ratio (INR) and liver-function tests to monitor treatment responses as in RCTs [40, 41]. It is also necessary to highlight that misspecification of time zero is the major source bias in observational studies, but not lack of randomization [33, 34]. Successful emulation of hypothetical and real RCTs using observational data has been conducted recently [67–69]. In this study, we assured exchangeability between DOAC users and warfarin users by adjusting for baseline and time-varying confounding [42]. In addition, we aligned the time when all inclusion and exclusion criteria met, start of treatment strategies, and start of follow-up. Correct time zero specification in observational studies removed immortal time bias and prevalent user bias [33, 34].

This study has several limitations. First, unmeasured confounding such as patient frailty, body mass index, or physician’s preference were not adjusted in the analysis. Frailty was found to be a predictor of OAC selection and adverse outcomes in previous studies [70]. However, these variables are not available in SEER-Medicare data and we did not quantify the magnitudes of these unmeasured confounding in our analysis. Second, although we used validated algorithms to define exposure, outcomes, and covariates, measurement bias may still persist in claims data. For example, we measured patients’ behavioral risk factors (i.e., alcohol use disorders in HAS-BLED score) using ICD codes [71]. In recent studies, CHA_2_DS_2_-VASc score did not performed well in predicting risk of stroke in cancer patients, and novel assessment tool should be developed and validated for this population [72, 73]. Thus, our study could not fully capture the potential candidates for OAC initiation at study entry because a threshold of CHA_2_DS_2_-VASc score ≥ 2 may not be applicable for patients with AFib and cancer [74]. However, we found no difference when patients with all levels of CHA_2_DS_2_-VASc scores were included in the sensitivity analysis. In addition, 12-month baseline period before AFib diagnosis was insufficient to capture patients’ baseline characteristics. It is also noticed that socioeconomic factors were available on an aggregate Census tract level. Third, the presence of extreme weights may increase the variability of the treatment effects, although we found consistent findings after truncating weights to 95th percentile [75]. Fourth, some analyses, especially subgroup analyses, may be underpowered or could not be performed due to small sample size, leading to unstable treatment effects and wide 95% CIs. Likewise, we could not conduct the analysis stratified by individual DOACs due to limited sample size. Prior studies suggested apixaban stood out among DOACs in reducing risk of stroke and bleeding [21, 38]. Fifth, our findings may not be generalizable to non-Medicare populations or those who developed cancer after AFib diagnosis.

## Conclusions

In this target trial emulation using linked cancer registry and administrative claims data, we found that DOACs are safe and effective alternatives to warfarin in the management of patients with AFib and cancer.

## Supplementary Information

Below is the link to the electronic supplementary material.

Supplementary file1 (DOCX 811 KB)

## Data Availability

This study used the linked SEER-Medicare database. The interpretation and reporting of these data are the sole responsibility of the authors. The authors acknowledge the efforts of the National Cancer Institute; the Centers for Medicare and Medicaid Services, Information Management Services (IMS), Inc.; and the Surveillance, Epidemiology, and End Results (SEER) Program tumor registries in the creation of the SEER-Medicare database. The collection of cancer incidence data used in this study was supported by the California Department of Public Health pursuant to California Health and Safety Code Sect. 103,885; Centers for Disease Control and Prevention’s (CDC) National Program of Cancer Registries, under cooperative agreement 1NU58DP007156; the National Cancer Institute’s Surveillance, Epidemiology and End Results Program under contract HHSN261201800032I awarded to the University of California, San Francisco, contract HHSN261201800015I awarded to the University of Southern California, and contract HHSN261201800009I awarded to the Public Health Institute.

## Code Availability

Available from authors upon request.
