# Supplementary material for: Effectiveness and Safety of Direct Oral Anticoagulants Versus Warfarin in Patients with Atrial Fibrillation and Cancer: A Target Trial Emulation from SEER-Medicare Database
Source: Cardiovasc Drugs Ther. 2024 Jun 7;39(4):823–35. doi: 10.1007/s10557-024-07589-7 (PMC12296962; doi:10.1007/s10557-024-07589-7)
Supplement: Supplementary file 1 — Supplementary file1 (DOCX 811 KB) [file 10557_2024_7589_MOESM1_ESM.docx]

**Effectiveness and Safety of Direct Oral Anticoagulant versus Warfarin in patients with Atrial Fibrillation and Cancer:
A target trial emulation from SEER-Medicare database**

Bang Truong, PhD^1^; Lori Hornsby, PharmD^2,^ Brent Fox, PharmD, PhD^1^; Chiahung Chou, PhD^1^; Jingyi Zheng, PhD^3^; Jingjing Qian, PhD^1^.

**Supplementary materials (tables, figures)**

**Table S1.** Algorithms to identify study components from SEER-Medicare data

| **Component** | **Code type** | **Codes** |
| --- | --- | --- |
| ***Eligibility criteria*** | | |
| AFib | ICD-9-CM | 427.31 or 427.32 |
|  | ICD-10-CM | I48.xx |
| Breast cancer | ICD-O-3 | C50.0-C50.9 |
| Lung cancer | ICD-O-3 | C34.0, C34.1, C34.2, C34.3, C34.8, C34.9, C33.9 |
| Prostate cancer | ICD-O-3 | C61.9 |
| Exclusion criteria |  |  |
| Valvular heart diseases | ICD-9-CM | 0932, 394, 395, 396, 3970, 3971, 240, 4241, 242, 4243, 7460, 7461, 7462, 7463, 7464, 7465, 7466, 99602, 99671, V422 |
|  | ICD-10-CM | I05, I06, I07, I08, I34, I35, I36, I37, I39, Q22, Q23, T820, T8201, T8202, T8203, T8209, T8222, T826, Z952, Z953, Z954 |
|  | ICD-9-CM PX | 351, 352, 3533, 3595, 3599 |
|  | ICD-10-PCS | 02RF, 02RG, 02RH, 02RJ, 02QF, 02QG, 02QH, 02QJ |
| Heart valve repair or replacement | ICD-9-CM | V433 |
| VTE | ICD-9-CM | 4151 ,453, V1251, V1255 |
|  | ICD-10-CM | I26, I80, I81, I82, Z8671 |
| Joint replacement | ICD-9-CM PX | 8151, 8152, 8154 |
|  | ICD-10-PCS | 0SR9019, 0SR901A, 0SR901Z, 0SR9029, 0SR902A, 0SR902Z, 0SR9039, 0SR903A, 0SR903Z, 0SR9049, 0SR904A, 0SR904Z, 0SR9069, 0SR906A, 0SR906Z, 0SR907Z, 0SR90EZ, 0SR90J9, 0SR90JA, 0SR90JZ, 0SR90KZ, 0SRA009, 0SRA00A, 0SRA00Z, 0SRA019, 0SRA01A, 0SRA01Z, 0SRA039, 0SRA03A, 0SRA03Z, 0SRA07Z, 0SRA0J9, 0SRA0JA, 0SRA0JZ, 0SRA0KZ, 0SRB019, 0SRB01A, 0SRB01Z, 0SRB029, 0SRB02A, 0SRB02Z, 0SRB039, 0SRB03A, 0SRB03Z, 0SRB049, 0SRB04A, 0SRB04Z, 0SRB069, 0SRB06A, 0SRB06Z, 0SRB07Z, 0SRB0EZ, 0SRB0J9, 0SRB0JZ, 0SRB0KZ, 0SRE009, 0SRE00A, 0SRE00Z, 0SRE019, 0SRE01A, 0SRE039, 0SRE03A, 0SRE03Z, 0SRE07Z, 0SRE0J9, 0SRE0JA, 0SRE0JZ, 0SRR019, 0SRR01A, 0SRR01Z, 0SRR039, 0SRR03A, 0SRR03Z, 0SRB0JA, 0SRR07Z, 0SRR0J9, 0SRR0JA, 0SRR0JZ, 0SRR0KZ, 0SRS019, 0SRE01Z, 0SRS01A, 0SRS01Z, 0SRS039, 0SRS03A, 0SRS03Z, 0SRS07Z, 0SRE0KZ, 0SRS0J9, 0SRS0JA, 0SRS0JZ, 0SRS0KZ, 0SRC069, 0SRC06A, 0SRC06Z, 0SRC07Z, 0SRC0EZ, 0SRC0J9, 0SRC0JA, 0SRC0JZ, 0SRC0KZ, 0SRC0L9, 0SRC0LA, 0SRC0LZ, 0SRC0M9, 0SRC0MA, 0SRC0MZ, 0SRC0N9, 0SRC0NA, 0SRC0NZ, 0SRD069, 0SRD06A, 0SRD06Z, 0SRD07Z, 0SRD0EZ, 0SRD0J9, 0SRD0JA, 0SRD0JZ, 0SRD0KZ, 0SRD0L9, 0SRD0LA, 0SRD0LZ, 0SRD0M9, 0SRD0MA, 0SRD0MZ, 0SRD0N9, 0SRD0NA, 0SRD0NZ, 0SRT07Z, 0SRT0J9, 0SRT0JA, 0SRT0JZ, 0SRT0KZ, 0SRU07Z, 0SRU0J9, 0SRU0JA, 0SRU0JZ, 0SRU0KZ, 0SRV07Z, 0SRV0J9, 0SRV0JA, 0SRV0JZ, 0SRV0KZ, 0SRW07Z, 0SRW0J9, 0SRW0JA, 0SRW0JZ, 0SRW0KZ |
| Renal impairment stage 5/ESRD | ICD-9-CM | 40301, 40311, 40391, 5855, 5856, V451, V56 |
|  | ICD-10-CM | I120, I1311, I132, N185, Y841, Z49, Z9115, Z992 |
|  | ICD-9-CM PX | 3995, 5498 |
|  | ICD-10-PCS | 3E1M39Z, 5A1D70Z, 5A1D80Z, 5A1D90Z |
| History of stroke/TIA | ICD-9-CM | 36231, 36232, 36233, 36234, 43301, 43311, 43321, 43331, 43381, 43391, 43401, 43411, 436, 430, 431, 43391, 435 |
|  | ICD-10-CM | H340, H341, H342, I63, I60, I61, I62, I63, G45, I6782, I6789 |
| Major surgery |  | Not included due to large number of codes |
| Intracranial/Spinal bleeding | ICD-9-CM | 430, 431, 432, 852, 853, |
|  | ICD-10-CM | I60, I61, I62, S064, S065, S066 |
| Intraocular bleeding | ICD-9-CM | 36281, 37923, 36361, 36362, |
|  | ICD-10-CM | H356, H3130, H3131, H431 |
| Retroperitoneal bleeding | ICD-9-CM | 56881 |
|  | ICD-10-CM | K661 |
| Atraumatic intra-articular bleeding | ICD-9-CM | 7191 |
|  | ICD-10-CM | M250 |
| Gastrointestinal bleeding | ICD-9-CM | 4560, 45620, 5301, 5307, 53082, 5310, 5311, 5312, 5313, 5314, 5315, 5316, 5317, 5319, 5320, 5321, 5322, 5323, 5324, 5325, 5326, 5327, 5329, 5330, 5331, 5332, 5333, 5334, 5335, 5336, 5337, 5339, 5340, 53400, 53401, 5341, 5342, 5343, 5344, 5345, 5346, 5347, 5349, 53500, 53501, 53510, 53511, 53520, 53521, 53530, 53531, 53540, 53541, 53550, 53551, 53560, 53561, 53783, 5780, 4551, 4552, 4554, 4555, 4556, 4557, 4558, 4559, 56200, 56201, 56202, 56203, 56210, 56211, 56212, 56213, 5693, 56985, 5781, 5789 |
|  | ICD-10-CM | I8501, I8511, K20, K210, K2211, K226, K250, K251, K252, K254, K255, K256, K260, K261, K262, K264, K265, K266, K270, K271, K272, K274, K275, K276, K280, K281, K282, K284, K285, K286, K2901, K2921, K2931, K2941, K2951, K2961, K2971, K2981, K2991, K31811, K920, K5521, K5701, K5711, K5721, K5731, K5741, K5751, K5753, K5781, K5791, K5793, K625, K640, K641, K642, K643, K644, K645, K648, K649, K921, K922 |
| ***Outcomes*** | | |
| Ischemic stroke (new diagnosis) | ICD-9-CM | 36231, 36232, 36233, 36234, 43301, 43311, 43321, 43331, 43381, 43391, 43401, 43411, 436 |
|  | ICD-10-CM | H340, H341, H342, I63 |
| Major bleeding | ICD-9-CM | 3361, 36361, 36362, 36372, 37632, 37742, 37923, 4230, 430, 431, 432, 56881, 7191, 72992, 852, 853, 86601, 86602, 86611, 86612 |
|  | ICD-10-CM | G9519, H0523, H3130, H3131, H3141, H431, H4702, I230, I312, I60, I61, I62, K661, M250, M7981, S064, S065, S066, S260, S3701, S3702, S3703, S3704, S3705, S3706 |
| VTE | ICD-9-CM | See eligibility criteria |
|  | ICD-10-CM |  |
| Intracranial bleeding | ICD-9-CM | See eligibility criteria |
|  | ICD-10-CM |  |
| GI bleeding | ICD-9-CM | See eligibility criteria |
|  | ICD-10-CM |  |
| Non-critical site bleeding | ICD-9-CM | 2800, 2851, 4551, 4552, 4554, 4555, 4556, 4557, 4558, 4559, 4560, 45620, 4590, 5301, 5307, 5310, 5311, 5312, 5313, 5314, 5315, 5316, 5317, 5319, 5320, 5321, 5322, 5323, 5324, 5325, 5326, 5327, 5329, 5330, 5331, 5332, 5333, 5334, 5335, 5336, 5337, 5339, 5340, 5341, 5342, 5343, 5344, 5345, 5346, 5347, 5349, 53500, 53501, 53510, 53511, 53520, 53521, 53530, 53531, 53540, 53541, 53550, 53551, 53560, 53561, 53783, 56200, 56201, 56202, 56203, 56210, 56211, 56212, 56213, 5693, 56985, 5780, 5781, 5789, 59381, 59970, 59971, 6236, 6238, 6266, 6268, 7847, 7848, 7863, D500, D62, I8501, I8511, K20, K210, K2211, K226, K250 |
|  | ICD-10-CM | K251, K252, K254, K255, K256, K260, K261, K262, K264, K265, K266, K270, K271, K272, K274, K275, K276, K280, K281, K282, K284, K285, K286, K2901, K2921, K2931, K2941, K2951, K2961, K2971, K2981, K2991, K31811, K5521, K5701, K5711, K5721, K5731, K5741, K5751, K5753, K5781, K5791, K5793, K625, K640, K641, K642, K643, K644, K645, K648, K649, K920, K921, K922, N897, N898, N921, N938, N939, R040, R041, R042, R310, R319, R58 |
| ***Covariates*** | | |
| CHF | ICD-9-CM | 39891, 40201, 40211, 40291, 40401, 40403, 40411, 40413, 40491, 40493, 4254, 4259, 428 |
|  | ICD-10-CM | I0981, I110, I130, I132, I425, I428, I50 |
| HTN | ICD-9-CM | 401, 402, 403, 404, 405 |
|  | ICD-10-CM | I10, I11, I12, I13, I14, I15, I16 |
| DM | ICD-9-CM | 250, 3572, 3620, 36641, |
|  | ICD-10-CM | E10, E11, E13 |
| Vascular diseases | ICD-9-CM | 410, 412, 4400, 4402, 4403, 4409, 4442, 4439, 44481 |
|  | ICD-10-CM | I21, I252, I700, I702, I703, I704, I705, I706, I707, I709, I742, I743, I744, I739, I745 |
| Renal diseases | ICD-9-CM | 0160, 0954, 1890, 1899, 2230, 23691, 2504, 2714, 2741, 28311, 403, 404, 4401, 4421, 4473,  5724, 580, 581, 582, 583, 584, 585, 586, 587, 588, 591, 6421, 6462, 75312, 75313, 75314, 75315, 75316, 75317, 75319, 7532, 7944, V420, V451, V56 |
|  | ICD-10-CM | A1811, A5275, C649, C689, D4100, E1129, E1029, E1121, E1021, E748, M1030, N200, D593, I120, I129, I1310, I130, I1311, I132, I1311, I701, I722, I773, K767, N00, N01, N02, N03, N04, N05, N06, N07, N08, N1330, O10419, O10411, O10412, O10413, O1042, O1043, O26839, O1214, O26831, O26832, O26833, Q613, Q612, Q6119, Q614, Q615,  Q6102, Q618, Q6239, Q6211, Q6212, Q6231, Q6210, Q6211, R944, Z940, Z992, Z9115, Z4931, Z4901, Z4902, Z4932 |
| Liver diseases | ICD-9-CM | 070, 07271, 09162, 1305, 571, 573, 7948 |
|  | ICD-10-CM | A5145, B0081, B15, B16, B17, B18, B19, B251, B2681, B581, B942, K70, K71, K72, K73, K74, K75, K76, K77, R94.5 |
| Bleeding disposition | ICD-9-CM | 430, 431, 432, 56881, 5997, 5307, 5310, 5312, 5314, 5316, 5320, 5322, 5324, 5326, 5330, 5332, 5334, 5336, 5340, 5342, 5344, 5346, 5693, 53501, 53511, 53521, 53531, 53541, 53551, 53561, 53571, 53783, 53784, 56202, 56203, 56212, 56213, 56985, 578, 7847, 7863, 6262, 7191, 37272, 459 |
|  | ICD-10-CM | I60, I61, I62, K661, R31, K226, K250, K252, K254, K256, K260, K262, K264, K266, K270, K272, K274, K276, K280, K282, K284, K286, K625, K2901, K2921, K2931, K2941, K2951, K2961, K2971, K2981, K2991, K31811, K3182, K5701, K5711, K5713, K5721, K5731, K5733, K5741, K5751, K5753, K5781, K5791, K5793, K5521, K920, K921, K922, R040, R042, N920, M250, M122, H113, R58 |
| Alcohol use disorders | ICD-9-CM | 291, 303, 3050, 3575, 4255, 5353, 5710, 5711, 5712, 5713, 7903 |
|  | ICD-10-CM | E8600, V113, F10, Z714 |
| Asthma/COPD | ICD-9-CM | 491, 492, 496, 49300, 49301, 49302, 49310, 49311, 49312, 49320, 49321, 49322, 49381, 49382, 49390, 49391, 49392 |
|  | ICD-10-CM | J41, J42, J43, J44, J4520, J4521, J4522, J4530, J4531, J4532, J4540, J4541, J4542, J4550, J4551, J4552, J45901, J45902, J45909, J45990, J45991, J45998 |
| Hematological disorders | ICD-9-CM | 280, 281, 282, 283, 284, 285, 286, 2871, 2873, 2874, 2875 |
|  | ICD-10-CM | D46, D50, D51, D52, D53, D55, D56, D57, D58, D59, D60, D61, D62, D63, D64 |
| Dementia | ICD-9-CM | 3310, 3311, 3312, 3317, 290, 2940, 2941, 2948, 797 |
|  | ICD-10-CM | G30, G310, G311, G312, G319, F02, F03, F04, R4181 |
| Depression | ICD-9-CM | 2962, 2963, 2965, 3004, 309, 311 |
|  | ICD-10-CM | F32, F33, F341, F43 |
| Thrombocytopenia | ICD-9-CM | 286, 287 |
|  | ICD-10-CM | D68, D69 |
| AKD | ICD-9-CM | 584 |
|  | ICD-10-CM | N17 |
| Peptic ulcer diseases | ICD-9-CM | 533, V1271, |
|  | ICD-10-CM | K27, Z8711 |
| Aspirin/NSAIDs | Generic drug name | ASPIRIN, CLOPIDOGREL, CELECOXIB, DICLOFENAC, DIFLUNISAL, ETODOLAC, FENOPROFEN, FLURBUPROFEN, IBUPROFEN, INDOMETHACIN, KETOPROFEN, KETOROLAC, MEFENAMIC, MELOXICAM, NABUMETONE, NAPROXEN, OXAPROZIN, PIROXICAM, SULINDAC, TOLMETIN |
| ACEI/ARB | Generic drug name | BENAZEPRIL, CAPTOPRIL, ENALAPRIL, FOSINOPRIL, LISINOPRIL, MOEXIPRIL, PERINDOPRIL, QUINAPRIL, RAMIPRIL, TRANDOLAPRIL LOSARTAN, IRBESARTAN, OLMESARTAN, VALSARTAN, TELMISARTAN, CANDESARTAN, AZILSARTAN |
| CCB | Generic drug name | AMLODIPINE, DILTIAZEM, FELODIPINE, ISRADIPINE, LEVAMLODIPINE, NIFEDIPINE, NISOLDIPINE, VERAPAMIL |
| BB | Generic drug name | ACEBUTOLOL, ATENOLOL, BETAXOLOL, BISOPROLOL, CARVEDILOL, LABETALOL, METOPROLOL , NADOLOL, NEBIVOLOL, PINDOLOL, PROPRANOLOL, TIMOLOL |
| Antiarrhythmic drugs | Generic drug name | QUINIDINE, PROCAINAMIDE, DISOPYRAMIDE, LIDOCAINE , MEXILETINE, FLECAINIDE, PROPAFENONE, AMIODARON, EDRONEDARONE, DOFETILIDE, SOTALOL, IBUTILID, DIGOXIN |
| Diuretics | Generic drug name | HYDROCHLOROTHIAZIDE, CHLOROTHIAZIDE, CHLORTHALIDONE, EPLERENONE, FUROSEMIDE, INDAPAMIDE, SPIRONOLACTONE , TORSEMIDE, METOLAZONE |
| Statins | Generic drug name | ATORVASTATIN, FLUVASTATIN, LOVASTATIN, PITAVASTATIN, PRAVASTATIN, ROSUVASTATIN, SIMVASTATIN |
| PPIs | Generic drug name | OMEPRAZOLE, ESOMEPRAZOLE, LANSOPRAZOLE, DEXLANSOPRAZOLE, PANTOPRAZOLE, RABEPRAZOLE |
| SSRI/SNRI | Generic drug name | CITALOPRAM, ESCITALOPRAM, FLUOXETINE, FLUVOXAMINE, PAROXETINE, SERTRALINE, VILAZODONE, DULOXETINE, VENLAFAXINE, LEVOMILNACIPRAN  DESVENLAFAXINE |

**Table S1.** Characteristics of patients with AFib and cancer who initiated oral anticoagulants in SEER-Medicare (2012-2019)

|  | DOAC initiators  (N=5371) | Warfarin initiators  (N=1778) | p-value |
| --- | --- | --- | --- |
| Demographics | | | |
| Index age (Mean, SD) | 77.32 (6.73) | 77.09 (6.50) | 0.2108 |
| Year of AFib diagnosis |  |  | <.0001 |
| 2012-2015 | 1478 (27.52) | 1175 (65.72) |  |
| 2016-2019 | 3893 (72.48) | 613 (34.28) |  |
| Female | 2883 (53.68) | 911 (50.95) | 0.0454 |
| Race/ethnicity |  |  | 0.6995 |
| Non-Hispanic White | 4660 (86.76) | 1558 (86.76) |  |
| Non-Hispanic Black | 274 (5.10) | 95 (5.31) |  |
| Others | 437 (8.14) | 135 (7.55) |  |
| Region |  |  | <.0001 |
| Midwest | 484 (9.01) | 266 (14.88) |  |
| Northeast | 2386 (44.42) | 761 (42.56) |  |
| South | 977 (18.19) | 275 (15.38) |  |
| West | 1524 (28.36) | 486 (27.18) |  |
| Medicaid eligible | 886 (16.50) | 321 (17.95) | 0.1541 |
| Urbanicity |  |  | <.0001 |
| Metropolitan | 4676 (87.06) | 1449 (81.04) |  |
| Micropolitan | 388 (7.22) | 191 (10.68) |  |
| Unknown/Missing | 307 (5.72) | 148 (8.28) |  |
| Socioeconomic status (Census Tract) | | | |
| Household median income (Median, IQR) | 64894.00  (46275.00-90291.00) | 58494.00  (44751.00-80924.00) | <.0001 |
| Percentage of residents living below poverty (Median, IQR) | 8.39 (4.56-15.64) | 9.51 (5.32-16.59) | <.0001 |
| Percentage of non-high school graduates (Median, IQR) | 9.00 (4.91-16.21) | 9.59 (5.55-16.31) | 0.0054 |
| Percentage of high school only (Median, IQR) | 26.20 (17.74-34.56) | 28.57 (20.35-36.05) | <.0001 |
| Percentage of some college education (Median, IQR) | 27.56 (22.31-33.01) | 29.05 (23.78-34.31) | <.0001 |
| Percentage of college education and above (Median, IQR) | 30.98 (17.82-47.72) | 26.27 (16.51-42.08) | <.0001 |
| Cancer characteristics |  |  |  |
| Time from cancer diagnosis to the onset of AFib (month, Median, IQR) | 32 (11-56) | 14 (2-37) | <.0001 |
| Time from the onset of AFib to treatment initiation (month, Median, IQR) | 0.26 (0.07-0.93) | 0.30 (0.10-0.89) | 0.0036 |
| Cancer type |  |  | <.0001 |
| Breast | 2182 (40.63) | 577 (32.27) |  |
| Lung | 1361 (25.34) | 705 (39.43) |  |
| Prostate | 1828 (34.03) | 506 (28.30) |  |
| Active cancer | 1399 (26.05) | 519 (29.03) | 0.0137 |
| Cancer grade |  |  | <.0001 |
| I | 814 (15.16) | 250 (13.98) |  |
| II | 1997 (37.18) | 586 (32.77) |  |
| III | 1614 (30.05) | 561 (31.38) |  |
| Others | 946 (17.61) | 391 (21.87) |  |
| Number of regional nodes examined |  |  | 0.0026 |
| <12 | 3378 (62.89) | 1046 (58.50) |  |
| ≥12 | 317 (5.90) | 112 (6.26) |  |
| Unknown/missing | 1676 (31.20) | 630 (35.23) |  |
| Tumor size |  |  | <.0001 |
| ≤2 cm | 1361 (25.34) | 384 (21.48) |  |
| 2-5 cm | 834 (15.53) | 280 (15.66) |  |
| >5 cm | 287 (5.34) | 140 (7.83) |  |
| Unknown/missing | 2889 (53.79) | 984 (55.03) |  |
| TMN classification |  |  |  |
| T stage |  |  | <.0001 |
| TX | 158 (2.94) | 70 (3.91) |  |
| T0 | 276 (5.14) | 59 (3.30) |  |
| T1 | 1761 (32.79) | 455 (25.45) |  |
| T2 | 1096 (20.41) | 349 (19.52) |  |
| T3 | 300 (5.59) | 128 (7.16) |  |
| T4 | 226 (4.21) | 92 (5.15) |  |
| Unknown/missing | 1554 (28.93) | 635 (35.51) |  |
| N stage |  |  | <.0001 |
| NX | 179 (3.33) | 62 (3.47) |  |
| N0 | 2877 (53.57) | 802 (44.85) |  |
| N1 | 354 (6.59) | 104 (5.82) |  |
| N2 | 303 (5.64) | 132 (7.38) |  |
| N3 | 104 (1.94) | 53 (2.96) |  |
| Unknown/missing | 1554 (28.93) | 635 (35.51) |  |
| M stage |  |  | <.0001 |
| M0 | 3446 (64.53) | 964 (53.83) |  |
| M1 | 349 (6.50) | 213 (10.89) |  |
| Unknown/missing | 1556 (28.97) | 636 (35.57) |  |
| Summary stage |  |  | <.0001 |
| In situ | 390 (7.26) | 91 (5.09) |  |
| Local | 3187 (59.34) | 954 (53.36) |  |
| Regional | 983 (18.30) | 361 (20.19) |  |
| Distant | 564 (10.50) | 287 (16.05) |  |
| Unknown/missing | 247 (4.60) | 95 (5.31) |  |
| Breast cancer-specific (N=2759) | | | |
| ER positive | 1350 (61.87) | 314 (54.42) | 0.0014 |
| PR positive | 1167 (53.48) | 269 (46.62) | 0.0005 |
| HER2 positive | 121(5.55) | 27 (4.68) | 0.0251 |
| Lung cancer-specific (N=2066) | | | |
| Histologic type |  |  | 0.7832 |
| Adenoma | 635 (46.66) | 324 (45.94) |  |
| NOS | 208 (15.28) | 110 (15.60) |  |
| Squamous | 409 (30.05) | 222 (31.49) |  |
| Others | 109 (8.01) | 49 (6.95) |  |
| Cancer treatment |  |  |  |
| Use of potentially interacting antineoplastic agents | 1482 (27.59) | 427 (23.88) | 0.0021 |
| Radiation | 219 (4.08) | 103 (5.76) | 0.0029 |
| Surgery | 33 (0.61) | 19 (1.06) | 0.0532 |
| Disease risk score |  |  |  |
| CHA_2_DS_2_-VASc |  |  | 0.1393 |
| 2 | 864 (16.44) | 294 (16.44) |  |
| 3 | 1505 (28.02) | 450 (25.17) |  |
| 4 | 1562 (29.08) | 544 (30.43) |  |
| 5 | 962 (17.91) | 316 (17.67) |  |
| ≥6 | 478 (8.90) | 184 (10.35) |  |
| HAS-BLED |  |  | 0.0048 |
| 1 | 544 (10.13) | 213 (11.91) |  |
| 2 | 2191 (40.79) | 651 (36.41) |  |
| 3 | 1709 (31.82) | 568 (31.77) |  |
| 4 | 714 (13.29) | 282 (15.77) |  |
| 5 | 184 (3.43) | 62 (3.47) |  |
| ≥6 | 29 (0.54) | 12 (0.67) |  |
| NCI Comorbidity score (Mean, SD) | 0.62 (0.57) | 0.70 (0.60) | <.0001 |
| Individual comorbidities |  |  |  |
| Asthma/COPD | 1766 (32.88) | 732 (40.94) | <.0001 |
| Hematological disorders | 1557 (29.36) | 516 (28.86) | 0.6856 |
| Dementia | 249 (4.64) | 55 (3.08) | 0.0046 |
| Depression | 791 (14.73) | 231 (12.92) | 0.0584 |
| Thrombocytopenia | 285 (5.31) | 97 (5.43) | 0.8465 |
| Acute kidney diseases | 243 (4.52) | 96 (5.37) | 0.1452 |
| Peptic ulcer diseases | 35 (0.65) | 18 (1.01) | 0.1293 |
| Medications |  |  |  |
| ACE inhibitors/ARBs | 3065 (57.07) | 985 (55.09) | 0.1442 |
| CCB | 1853 (34.50) | 605 (33.84) | 0.6088 |
| Beta blockers | 2389 (44.48) | 807 (45.13) | 0.6296 |
| Antiarrhythmic medications | 297 (5.53) | 124 (6.94) | 0.0068 |
| Diuretics | 2145 (39.94) | 733 (41.00) | 0.4290 |
| Statin | 2906 (54.11) | 938 (52.46) | 0.1804 |
| PPIs | 1446 (26.92) | 450 (25.17) | 0.1453 |
| SSRIs/SNRIs | 967 (18.00) | 280 (15.66) | 0.0236 |

**Table S2**. Distributions of time-varying weights before and after truncation

| Variable | Mean | Minimum | Maximum | 99th Pctl | Std Dev | Median | 25th Pctl | 75th Pctl |
| --- | --- | --- | --- | --- | --- | --- | --- | --- |
| ITT analysis | | | | | | | | |
| Stabilized weights | 1.43 | 0.02 | 4377.91 | 5.99 | 21.34 | 0.99 | 0.93 | 1.02 |
| Truncated stabilized weights (99^th^ percentile) | 1.08 | 0.02 | 5.99 | 5.99 | 0.67 | 0.99 | 0.93 | 1.02 |
| PP analysis |  |  |  |  |  |  |  |  |
| Stabilized weights | 1.51 | 0.02 | 6130.76 | 6.06 | 28.59 | 0.99 | 0.93 | 1.02 |
| Truncated stabilized weights (99^th^ percentile) | 1.08 | 0.02 | 6.06 | 6.06 | 0.68 | 0.99 | 0.93 | 1.02 |

ITT Intention-to-treat, PP Per-protocol, Pctl percentile

**Table S3**. Subgroup analyses for comparative effectiveness and safety between DOACs and warfarin in patients with atrial fibrillation and cancer

|  | Event (n, %) | | Adjusted HR (95% CI) |
| --- | --- | --- | --- |
|  | **DOACs** | **Warfarin** |  |
| Cancer type | | | |
| *Breast (N=2759)* |  |  |  |
| Stroke | 17 (0.78) | -- | 0.95 (0.42-2.19) |
| Major bleeding | 11 (0.50) | -- | 0.88 (0.28-2.72) |
| VTE | -- | -- | **0.17 (0.07-0.44)** |
| Intracranial bleeding | -- | -- | 1.04 (0.29-3.66) |
| Gastrointestinal bleeding | 48 (2.20) | 13 (2.25) | 0.91 (0.55-1.51) |
| Non-critical site bleeding | 60 (2.75) | 20 (3.47) | 0.78 (0.50-1.15) |
| *Lung (N=2066)* |  |  |  |
| Stroke | 17 (1.25) | -- | 1.07 (0.56-2.02) |
| Major bleeding | -- | -- | 0.57 (0.27-1.19) |
| VTE | 18 (1.32) | 12 (1.70) | 1.46 (0.81-2.61) |
| Intracranial bleeding | - | - | 0.49 (0.22-1.09) |
| Gastrointestinal bleeding | 65 (4.78) | 25 (3.55) | 0.99 (0.67-1.47) |
| Non-critical site bleeding | 72 (5.53) | 39 (5.53) | 0.73 (0.52-1.02) |
| *Prostate (N =2334)* |  |  |  |
| Stroke | 11 (0.60) | -- | - |
| Major bleeding | 14 (0.77) | -- | - |
| VTE | -- | -- | - |
| Intracranial bleeding | 11 (0.60) | -- | 2.18 (0.49-9.68) |
| Gastrointestinal bleeding | 39 (2.13) | 24 (4.74) | **0.67 (0.42-0.98)** |
| Non-critical site bleeding | 53 (2.90) | 29 (5.73) | **0.66 (0.44-0.99)** |
| Cancer status |  |  |  |
| *Active cancer (N=1918)* |  |  |  |
| Stroke | -- | -- | 1.34 (0.41-4.41) |
| Major bleeding | -- | -- | 1.20 (0.57-2.77) |
| VTE | 12 (0.86) | -- | 1.12 (0.50-2.50) |
| Intracranial bleeding | -- | -- | 0.74 (0.28-1.94) |
| Gastrointestinal bleeding | 50 (3.57) | 19 (3.66) | 0.72 (0.47-1.12) |
| Non-critical site bleeding | 61 (4.36) | 29 (5.59) | 0.58 (0.58-1.83) |
| *Inactive (N=5241)* |  |  |  |
| Stroke | 35 (0.88) | 12 (0.95) | 1.12 (0.63-1.98) |
| Major bleeding | 26 (0.65) | -- | 0.85 (0.44-1.65) |
| VTE | 20 (0.50) | 16 (1.26) | **0.58 (0.34-0.99)** |
| Intracranial bleeding | 22 (0.55) | -- | 0.81 (0.40-1.63) |
| Gastrointestinal bleeding | 103 (2.59) | 43 (3.39) | 0.76 (0.56-1.05) |
| Non-critical site bleeding | 124 (3.12) | 59 (4.65) | **0.62 (0.48-0.81)** |
| Cancer stage | | | |
| *Local (N=4333)* |  |  |  |
| Stroke | 22 (0.66) | -- | 1.53 (0.69-3.42) |
| Major bleeding | 19 (0.57) | -- | 1.69 (0.65-4.39) |
| VTE | 14 (0.42) | -- | 0.91 (0.44-2.00) |
| Intracranial bleeding | 16 (0.48) | -- | 1.19 (0.44-3.18) |
| Gastrointestinal bleeding | 88 (2.64) | 36 (3.57) | **0.69 (0.49-0.98)** |
| Non-critical site bleeding | 110 (3.30) | 52 (5.16) | **0.58 (0.44-0.78)** |
| *Regional (N=1414)* |  |  |  |
| Stroke | 11 (1.07) | -- | - |
| Major bleeding | -- | -- | **0.41 (0.17-0.97)** |
| VTE | -- | -- | 0.88 (0.32-2.42) |
| Intracranial bleeding | -- | -- | **0.36 (0.15-0.88)** |
| Gastrointestinal bleeding | 35 (3.39) | 12 (3.17) | 0.76 (0.44-1.32) |
| Non-critical site bleeding | 43 (4.17) | 17 (4.45) | 0.77 (0.47-1.24) |
| *Distant (N=899)* |  |  |  |
| Stroke | -- | -- | 0.92 (0.27-3.12) |
| Major bleeding | -- | -- | 0.91 (0.24-3.35) |
| VTE | -- | -- | 0.58 (0.20-1.66) |
| Intracranial bleeding | -- | -- | - |
| Gastrointestinal bleeding | 24 (4.03) | 11 (3.69) | 0.96 (0.47-1.96) |
| Non-critical site bleeding | 26 (4.37) | 15 (5.03) | 1.19 (0.73-1.95) |
| Tumor grade | | | |
| *Grade I (N=1064)* |  |  |  |
| Stroke | -- | -- | - |
| Major bleeding | -- | -- | - |
| VTE | -- | -- | - |
| Intracranial bleeding | -- | -- | - |
| Gastrointestinal bleeding | 21 (2.58) | -- | 0.70 (0.37-1.32) |
| Non-critical site bleeding | 25 (3.07) | -- | 0.74 (0.39-1.42) |
| *Grade II (N=2583)* |  |  |  |
| Stroke | 20 (1.00) | -- | 2.24 (0.80-6.29) |
| Major bleeding | 11 (0.55) | -- | 0.98 (0.32-3.10) |
| VTE | -- | -- | **0.42 (0.19-0.98)** |
| Intracranial bleeding | -- | -- | 1.31 (0.33-3.96) |
| Gastrointestinal bleeding | 49 (2.45) | 24 (4.10) | 0.72 (0.45-1.14) |
| Non-critical site bleeding | 61 (3.05) | 31 (5.29) | **0.61 (0.41-0.90)** |
| *Grade III (N=2175)* |  |  |  |
| Stroke | -- | -- | 1.12 (0.34-3.69) |
| Major bleeding | -- | -- | **0.28 (0.12-0.67)** |
| VTE | -- | -- | 0.60 (0.24-1.48) |
| Intracranial bleeding | -- | -- | **0.22 (0.08-0.60)** |
| Gastrointestinal bleeding | 48 (2.97) | 22 (3.92) | 0.74 (0.47-1.16) |
| Non-critical site bleeding | 57 (3.53) | 27 (4.81) | 0.73 (0.48-1.09) |

-: suppressed due to cell size <11
VTE Venous Thromboembolism, DOACs Direct Oral Anticoagulants, HR Hazard Ratio, CI Confidence Interval

**Table S4**. Sensitivity analysis for comparative effectiveness and safety between DOACs and warfarin in patients with atrial fibrillation and cancer

|  | Event (n, %) | | Adjusted HR (95% CI) |
| --- | --- | --- | --- |
|  | **DOACs** | **Warfarin** |  |
| Sensitivity analysis 1 (N=7934) |  |  |  |
| Stroke | 51 (0.86) | 16 (0.80) | 1.16 (0.73-1.85) |
| Major bleeding | 36 (0.61) | 16 (0.80) | 0.82 (0.51-1.34) |
| VTE | 34 (0.57) | 22 (1.10) | 0.75 (0.49-1.15) |
| Intracranial bleeding | 29 (0.47) | 14 (0.70) | 0.71 (0.42-1.20) |
| Gastrointestinal bleeding | 169 (2.84) | 69 (3.46) | 0.79 (0.62-1.00) |
| Non-critical site bleeding | 204 (3.03) | 99 (4.97) | **0.63 (0.52-0.78)** |
| Sensitivity analysis 2 (N=7558) |  |  |  |
| Stroke | 46 (0.81) | 15 (0.79) | 1.24 (0.75-2.05) |
| Major bleeding | 35 (0.62) | 14 (0.74) | 0.91 (0.54-1.52) |
| VTE | 35 (0.62) | 22 (1.16) | 0.74 (0.48-1.15) |
| Intracranial bleeding | 28 (0.49) | 12 (0.63) | 0.81 (0.46-1.41) |
| Gastrointestinal bleeding | 155 (2.74) | 63 (3.32) | **0.77 (0.60-0.99)** |
| Non-critical site bleeding | 187 (3.30) | 92 (4.85) | **0.61 (0.50-0.76)** |
| Sensitivity analysis 3 (N=7159) |  |  |  |
| Stroke | 89 (1.65) | 31 (1.73) | 1.21 (0.98-1.50) |
| Major bleeding | 69 (1.28) | 34 (1.89) | 0.74 (0.49-1.12) |
| VTE | 54 (1.00) | 33 (1.84) | **0.80 (0.66-0.98)** |
| Intracranial bleeding | 60 (1.12) | 27 (1.50) | 0.81 (0.51-1.28) |
| Gastrointestinal bleeding | 248 (4.61) | 97 (5.40) | 0.90 (0.71-1.14) |
| Non-critical site bleeding | 301 (5.60) | 135 (7.52) | **0.64 (0.56-0.74)** |
| Sensitivity analysis 4 (N=6266) |  |  |  |
| Stroke | 37 (0.77) | 11 (0.74) | 1.68 (0.88-3.24) |
| Major bleeding | 30 (0.63) | -- | 0.88 (0.49-1.59) |
| VTE | 24 (0.50) | 14 (0.94) | 0.64 (0.38-1.07) |
| Intracranial bleeding | 24 (0.50) | -- | 0.67 (0.37-1.24) |
| Gastrointestinal bleeding | 129 (2.70) | 51 (3.42) | **0.69 (0.52-0.91)** |
| Non-critical site bleeding | 159 (3.33) | 73 (4.90) | **0.59 (0.47-0.76)** |
| Sensitivity analysis 5 (N=6777) |  |  |  |
| Stroke | 41 (0.81) | 14 (0.83) | 1.20 (0.72-2.01) |
| Major bleeding | 33 (0.65) | 14 (0.83) | 0.84 (0.51-1.41) |
| VTE | 27 (0.53) | 20 (1.18) | 0.66 (0.42-1.05) |
| Intracranial bleeding | 27 (0.53) | 12 (0.71) | 0.76 (0.44-1.03) |
| Gastrointestinal bleeding | 145 (2.85) | 59 (3.47) | 0.77 (0.60-1.01) |
| Non-critical site bleeding | 176 (3.46) | 84 (4.97) | **0.63 (0.51-0.79)** |
| Sensitivity analysis 6 (N=7159) |  |  |  |
| Stroke | 45 (0.84) | 15 (0.84) | 1.17 (0.66-2.06) |
| Major bleeding | 35 (0.65) | 14 (0.78) | 0.84 (0.47-1.50) |
| VTE | 32 (0.60) | 20 (1.12) | 0.59 (0.35-1.00) |
| Intracranial bleeding | 28 (0.52) | 12 (0.67) | 0.81 (0.43-1.54) |
| Gastrointestinal bleeding | 153 (2.58) | 62 (3.47) | 0.80 (0.60-1.06) |
| Non-critical site bleeding | 185 (3.44) | 88 (4.92) | **0.67 (0.53-0.86)** |

-: suppressed due to cell size <11
VTE Venous Thromboembolism, DOACs Direct Oral Anticoagulants, HR Hazard Ratio, CI Confidence Interval

*Sensitivity analysis 1. Using a grace period of 6 months for OAC initiation
Sensitivity analysis 2. Including individuals with all levels of baseline CHA_2_DS_2_-VASc score
Sensitivity analysis 3. Extending maximum follow-up to 36 months
Sensitivity analysis 4. Excluding patients with metastatic cancer at baseline
Sensitivity analysis 5. Excluding patients with thrombocytopenia at baseline
Sensitivity analysis 6. Truncating stabilized weights at 95^th^ percentile*

**
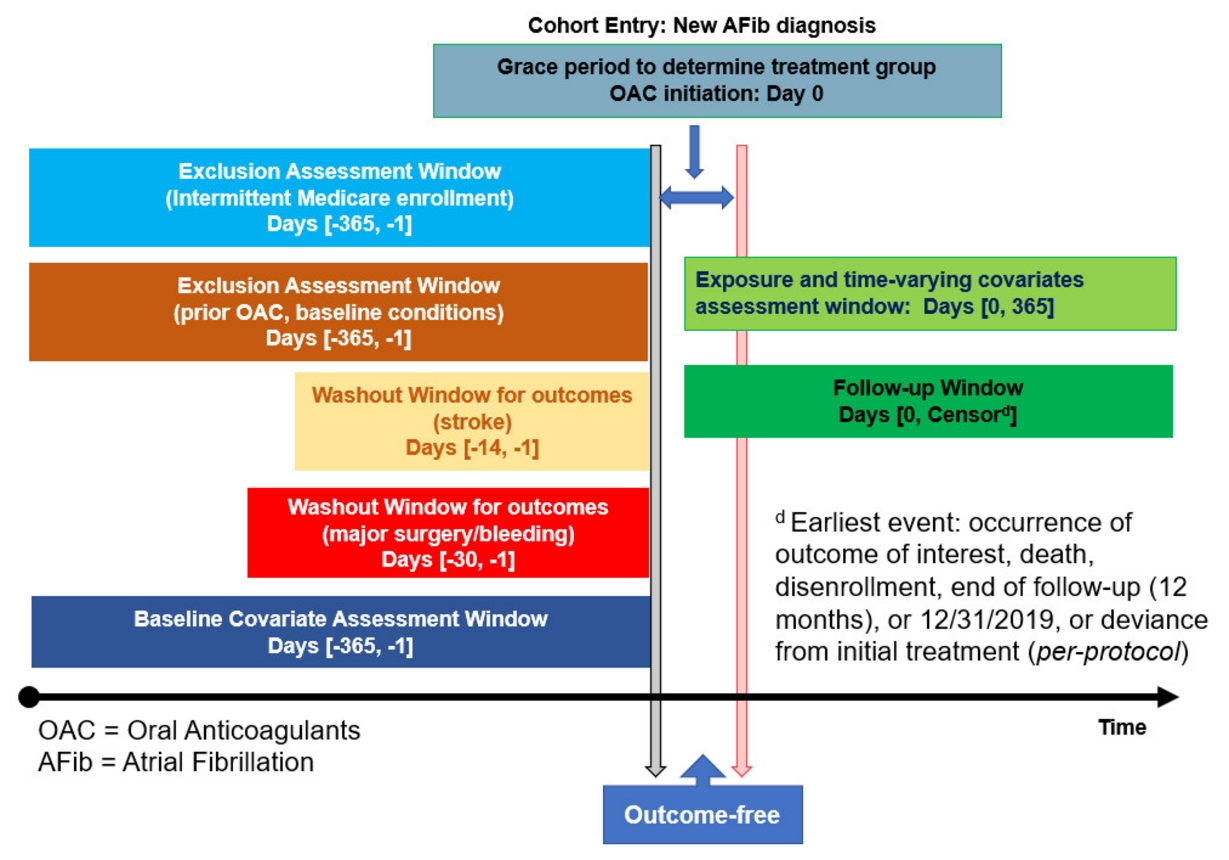
**

**Figure S1**. Visualization of study timeline

**Technical appendix^1-6^**

*1. ITT analysis*

The treatment is estimated effect via a 12-month risk of developing a specific outcome among those who initially started DOACs compared with warfarin.

First, we computed the probability of initiating DOACs or warfarin, conditioning on baseline covariates (A=1 for DOACs, A=0 for warfarin)

$logit(\Pr\left[ A=1 | L \right])$ = $\alpha_{0}$ + $\alpha_{1}^{T}L_{0}$

The stabilized weights (SW) of being assigned to either treatment were estimated by ${SW}^{A}$ = $\frac{f[A]}{f[A|L]}$. Therefore, the stabilized weight for treatment A=1: ${SW}^{A}$ = $\frac{\hat{Pr}[A=1]}{\hat{Pr}[A=1|L]}$ and for A=0: ${SW}^{A}$ = $\frac{1-\hat{Pr}[A=1]}{1-\hat{Pr}[A=1|L]}$.

We also applied inverse-probability weights to this model to adjust for potential selection bias:

$logit(\Pr\left[ C=0 | A, L \right])$ = $\pi_{0}$ + $\tau_{1}^{T}L_{0}+ \pi_{1}A$ (numerator)

$logit(\Pr\left[ C=0 | A, L \right])$ = $\tau_{0}$ + $\tau_{1}^{T}L_{0}+ \tau_{2}A$ (denominator)

The stabilized weights for censoring due to loss to follow-up is: ${SW}^{LFU}$ = $\frac{\hat{Pr}[LFU=0|A]}{\hat{Pr}[LFU=0|L,A]}$. Under the assumptions of no unmeasured confounding given the included covariates and a low monthly risk of the outcome within levels of those covariates, the hazards ratios (HR) for developing the outcomes if all eligible individuals had been treated with DOACs (a=1) versus all eligible individuals had been treated with warfarin (a=0) can be estimated by the marginal structural model^4^ below:

$logit(\Pr\left[ Y_{t+1}^{a}=1 | Y_{t}^{a}=0 \right])$ = $\gamma_{0}$ +$\gamma_{2}a$ + $\gamma_{3}at+$ $\gamma_{4}at^{2}$

Next, we organized each persons’ data in discrete-time (person-month) format (month 1 to 12) and fitted a weighted pooled logistic regression containing the outcome Y, and treatment A, and a function of time (time and its quadratic terms), weighted for SW calculated in the previous step. SWs were truncated at 99^th^ percentile to remove extreme weights.

$$\mathrm{logit} \left( \Pr\left[ Y_{t+1}=1|A_{0},L_{0}, \bar{Y}_{t}=0 \right] \right)=\alpha_{0,t}+\alpha_{1}A_{0}+{\alpha_{2}}^{T}L_{0}$$

The exponentiated coefficient of treatment indicator ($e^{\beta_{2}})$ is the unbiased estimate of ITT HR.

*2. PP analysis*

We arranged the data in person-month format and calculate subject-specific time-varying non-stabilized inverse-probability weights.^7,8^ The denominator of this weight at time *t* is the probability that an individual received his/her observed treatment history given covariate history by *t*. The application of these weights creates a pseudo-population in which treatment is independent of the measured confounders at all time points. To estimate the denominator, we fitted two separate models to allow the probabilities to differ according to prior treatment status. The first model was fit to person-months who received treatment A=0 in the previous month (*i.e.,* $A_{k-1}=0$)

$$\mathrm{logit}\left( \Pr\left[ A_{k}=1 |{A_{k-1}=0,\bar{L}}_{k},\overline{Y}_{k-1}=0 \right] \right)=\eta_{0,t}+\eta_{1}^{T}L_{0}+\eta_{2}^{T}L_{k}$$

Then, we fit the second model for person-months who received treatment A=1 in the previous month (*i.e.,* $A_{k-1}=1$):

$$\mathrm{logit}\left( \Pr\left[ A_{k}=1 {{|A}_{k-1}=1,\bar{L}}_{k},\overline{Y}_{k-1}=0 \right] \right)=\theta_{0,t}+\theta_{1}^{T}L_{0}+\theta_{2}^{T}L_{k}$$

where covariate history $\bar{L}_{k}$ was summarized by baseline $L_{0}$ and the most recent measurement of $L_{k}$. Then, the weights for censoring due to switching treatment is computed by

$$W_{t}^{A}=\prod_{k=0}^{t} \frac{1}{f(A_{k}|\bar{A}_{k-1},\bar{L}_{k},\overline{Y}_{k-1}=0)}$$

The total weights are the product of time-varying non-stabilized inverse-probability weights for censoring due to treatment switching, time-varying weights censoring due to lost to follow-up

To obtain the treatment effect, we fitted a pooled logistic regression to the censored data, weighted for total weights to adjust for time-varying confounding. We truncated weights at their 99^th^ percentile to prevent extreme weights. Under the same assumptions described in the previous section, the exponentiated coefficient of the treatment indicator (*i.e.,* ${exp(\beta}_{2})$) validly estimates the per-protocol HR.

$\mathrm{logit} \left( \Pr\left[ Y_{t+1}=1|A_{0},L_{0}, \bar{Y}_{t}=0,\bar{C}_{t+1}=0 \right] \right)=\beta_{0}$ + $\beta_{1}^{T}L_{0}$ +$\beta_{2}$*A*

To estimate the weighted survival curves and standardize the curves to baseline distribution of covariates, we fitted a similar model with an interaction term between treatment regimen A and f(t) – a function of time and time squared to allow for non-proportional hazards. The model was then used to predict outcomes of interest under each treatment strategy $A=a$. The details for creating standardized survival curves can be found in published studies.^9^

**References**

1. Faries D, Zhang X, Kadziola Z. *Real World Health Care Data Analysis: Causal Methods and Implementation Using SAS®.* SAS Institute; 2020.

2. Cain LE, Saag MS, Petersen M, et al. Using observational data to emulate a randomized trial of dynamic treatment-switching strategies: an application to antiretroviral therapy. *International Journal of Epidemiology.* 2016;45(6):2038-2049.

3. Hernán MA. How to estimate the effect of treatment duration on survival outcomes using observational data. *BMJ (Clinical research ed).* 2018;360:k182-k182.

4. Hernán MA, Robins JM. *Causal Inference: What If.* Boca Raton: Chapman & Hall/CRC; 2020.

5. Lyu H, Yoshida K, Zhao SS, et al. Delayed Denosumab Injections and Fracture Risk Among Patients With Osteoporosis : A Population-Based Cohort Study. *Ann Intern Med.* 2020;173(7):516-526.

6. Murray EJ, Hernán MA. Adherence adjustment in the Coronary Drug Project: A call for better per-protocol effect estimates in randomized trials. *Clin Trials.* 2016;13(4):372-378.

7. Dickerman BA, García-Albéniz X, Logan RW, Denaxas S, Hernán MA. Emulating a target trial in case-control designs: an application to statins and colorectal cancer. *International Journal of Epidemiology.* 2020;49(5):1637-1646.

8. Yland JJ, Chiu YH, Rinaudo P, Hsu J, Hernán MA, Hernández-Díaz S. Emulating a target trial of the comparative effectiveness of clomiphene citrate and letrozole for ovulation induction. *Hum Reprod.* 2022.

9. Murray EJ, Caniglia EC, Petito LC. Causal survival analysis: A guide to estimating intention-to-treat and per-protocol effects from randomized clinical trials with non-adherence. *Research Methods in Medicine & Health Sciences.* 2020;2(1):39-49.
